# A New Method to Address Unmet Needs for Extracting Individual Cell Migration Features from a Large Number of Cells Embedded in 3D Volumes

**DOI:** 10.1371/journal.pone.0022263

**Published:** 2011-07-15

**Authors:** Ivan Adanja, Véronique Megalizzi, Olivier Debeir, Christine Decaestecker

**Affiliations:** 1 Laboratory of Image Synthesis and Analysis (LISA), Faculty of Applied Science, Université Libre de Bruxelles (U.L.B.), Brussels, Belgium; 2 Laboratory of Toxicology, Faculty of Pharmacy, Université Libre de Bruxelles (U.L.B.), Brussels, Belgium; City of Hope National Medical Center and Beckman Research Institute, United States of America

## Abstract

**Background:**

In vitro cell observation has been widely used by biologists and pharmacologists for screening molecule-induced effects on cancer cells. Computer-assisted time-lapse microscopy enables automated live cell imaging in vitro, enabling cell behavior characterization through image analysis, in particular regarding cell migration. In this context, 3D cell assays in transparent matrix gels have been developed to provide more realistic in vitro 3D environments for monitoring cell migration (fundamentally different from cell motility behavior observed in 2D), which is related to the spread of cancer and metastases.

**Methodology/Principal Findings:**

In this paper we propose an improved automated tracking method that is designed to robustly and individually follow a large number of unlabeled cells observed under phase-contrast microscopy in 3D gels. The method automatically detects and tracks individual cells across a sequence of acquired volumes, using a template matching filtering method that in turn allows for robust detection and mean-shift tracking. The robustness of the method results from detecting and managing the cases where two cell (mean-shift) trackers converge to the same point. The resulting trajectories quantify cell migration through statistical analysis of 3D trajectory descriptors. We manually validated the method and observed efficient cell detection and a low tracking error rate (6%). We also applied the method in a real biological experiment where the pro-migratory effects of hyaluronic acid (HA) were analyzed on brain cancer cells. Using collagen gels with increased HA proportions, we were able to evidence a dose-response effect on cell migration abilities.

**Conclusions/Significance:**

The developed method enables biomedical researchers to automatically and robustly quantify the pro- or anti-migratory effects of different experimental conditions on unlabeled cell cultures in a 3D environment.

## Introduction

The recent advances and developments in microscopy, cell labeling and time-lapse imaging technologies, now allow dynamic monitoring of cells or molecules in 3D environments (*in vitro* and *in vivo*). The purpose of a given biological assay guides the choice of the imaging approach. Migration of unlabeled live cells can be observed in 3D gels *in vitro* under contrast enhancing microscopy [1], [2], digital holography microscopy [3], [4] or optical coherence tomography [5]. Other imaging techniques (such as fluorescence-based microscopy) that require cell labeling allow the study of cell migration as well as the analysis of dynamic cellular and molecular events inside living cells *in vitro*. These techniques include wide-field fluorescence microscopy combined with adapted deconvolution methods [6], [7], confocal microscopy [8], [9], [10] and multiphoton techniques [9], [11], [12]. *In vitro* tests are generally used to provide a range of initial information and *in vivo* tests, which are both more difficult to perform and more time- and money-consuming, are preferably used as the ultimate stage to confirm information provided by *in vitro* assays. To this end, some imaging techniques, such as multiphoton [13] and magnetic resonance imaging (MRI) [14], have been adapted to small animal research and enables *in vivo* long-term tracking of labeled cells in living animal.

While a large number of these studies focus on the visualization of real-time behavior of cells or molecular events, only few of them present specific image analysis methods adapted to their imaging technique in order to extract quantitative information. As detailed below the present paper focuses on the quantitative characterization of cell migration *in vitro* using 3D cell assays in transparent matrix gels for the purpose of screening anti-migratory drugs on cancer cells. In the following introductory sections we present the needs dictated by this drug screening application and a comparative analysis of automated 3D cell tracking methods (involving both unlabeled and labeled cells), highlighting their advantages and drawbacks regarding those needs.

### Needs of tools for automated 3D cell tracking

*In vitro* cell observation has been widely used by biologists and pharmacologists for screening molecule-induced effects on cancer cells. In this context, 3D cell assays in transparent matrix gels have been developed to provide more realistic *in vitro* 3D environments for monitoring cell behavior and cell migration in particular [15]–[18].

In this paper we propose an improved automated tracking method that is designed to robustly and individually follow a large number of unlabeled cells in 3D gels observed under phase-contrast microscopy. The method automatically detects and tracks individual cells evolving in a sequence of acquired volumes, using a template matching filtering method that in turn allows for robust detection and mean-shift tracking. The resulting trajectories quantify cell migration through statistical analysis of 3D trajectory descriptors.

This kind of information is important because cancer cell migration is related to the spread of cancer and metastasis and is an actual target in anti-cancer drug development. Confirming the impact of environment on cell behavior, comparative observations of 2D and 3D cell cultures have shown that cells can exhibit different phenotypes in terms of gene expression, proliferation, shape, locomotion and multi-cellular organization [19], [20]. More recently an important study [21] showed that the way cells move inside a 3D environment is fundamentally different from the motility behavior observed in 2D (i.e., in conventional flat culture dishes), even if the support is coated by the same matrix than that used to constitute 3D gels. This later study highlighted that the shape and mode of movement for cells in 2D are merely an artifact of their environment, which could produce misleading results when testing the effects of different drugs on cell migration. This evidence thus shows that observing 2D cell migration behavior may be a poor indicator of the abilities of the same cells to move in their natural 3D environments.

While there exist many 2D assays to monitor cell motility, markedly fewer studies consider 3D experiments [15], [16], [22]. The focus of the present study is the quantification of cell migration in 3D matrix through tracking individual cells. Our goal was to create convenient tools for biologists and pharmacologists that allow them to automatically extract statistically robust 3D migration descriptors of the observed cell population from individual cell trajectories. The comparison between the trajectory descriptors of cell populations submitted to different experimental conditions or treatments allows to establish whether a statistically significant pro- or anti-migratory effect is observed. Our method is adapted to assays where cells are relatively sparsely seeded, as we are interested in measuring individual cells' motion without previously labeling cells, as detailed below. Our developments do not address 3D assays that involve multicellular aggregates, such as cell spheroids invading a matrix gel or other 3D substrates [23]. These latter assays usually consist of end-point studies evaluating global invasion distances after a time period (1 or several days) but do not consider individual cell migration. As previously discussed in [15], [16], the analysis of individual cell trajectories offers a number of advantages such as (i) the distinction between cell migration and cell growth (both involved in the invading process), (ii) the identification of subpopulations of cells presenting different migratory characteristics and/or different responses to a treatment, and (iii) the possibility of detecting preferential directions followed by moving cells (e.g., in a chemo-attraction assay). It should be also noted that collective cell motion can be efficiently quantified without identifying single cells, e.g., by optical flow techniques [24].

As detailed in the next section, a few methods based on fluorescent microscopy were developed to track a reduced number of cells migrating in 3D gels. However, the needs of the application dictate a large amount of data. As a 3D cell culture assay is more difficult to set-up and observe than a 2D cell culture, it is preferable to exploit as much available information as possible from each 3D migration assay. Many cells thus need to be individually and simultaneously tracked to provide statistically valid descriptors. Consequently, large volumes have to be observed, requiring relatively low magnification (e.g., 10× objective) and deep gel scanning (e.g., several hundred µm) through the acquisition of 3D image stacks. In this context, interactive tracking solutions are too tedious and fully automatic methods are required. To be robust, the automatic method should overcome the problems usually encountered in tracking a large amount of cells, such as cells touching, dividing, entering and exiting the observed field. While a number methods were developed for tracking large populations of cells in 2D environments, comparatively few automatic 3D tracking methods are proposed, as described in the following section. A comprehensive review of the literature on both 2D and 3D cell tracking can be found in [15], [16], [22], [25].

### Related studies

Usually, live cells are observed under either fluorescence-based microscopy or contrast enhancing microscopy (such as phase-contrast). Fluorescence-based microscopy requires cell labeling and more complex and costly microscopes and image acquisition systems. Various difficulties are also inherent to this technology such as cell toxicity due to repetitive fluorophore excitations, marker fading and problematic marker transfer during cell division [16]. These difficulties motivated us to focus our own developments on unlabeled cells observed under phase-contrast microscopy. This option was little investigated in previous studies (see Table 1)

**Table 1:** Related works on 3D cell tracking.

Authors	Cell labeling/microscopy type	Magnification	Tracking technique	Developed for large population tracking
Demou et al. 2002 [1]	Unlabeled/Hoffman m.	10×	Threshold-based detection and nearest neighbour association	Yes
Rabut et al. 2004 [27]	Labeled/unspecified fluorescence m.	63×	Center of mass adjustment guiding on-line microscope stage centering	No
Dufour et al. 2005 [9]	Labeled/two-photon and confocal m.	N/A (apparently large from illustrations)	Adapted level set	No
Chen et al. 2009 [12]	Labeled/two-photon m.	N/A (field size: 256×256 µm)	Mean-shift segmentation and multihypothesis association method	No
Dzyubachyk et al. 2010 [10]	Labeled/confocal m.	10×/63×	Adapted level set	No
Adanja et al. 2010 [2]	Unlabeled/phase-contrast m.	10×	Pattern correlation and mean-shift tracking	Yes

Details of the analysis are provided in File S1.

Table 1 summarizes the few studies describing automatic 3D cell tracking methods which are related to 3D cell culture assays. It does not include works related to 3D reconstruction of cells moving on 2D substrates (such as [26]). In this Table, we consider each method using different criteria in relation with the needs dictated by the drug screening application in focus in the present paper (details on this analysis are provided in File S1).

As indicated in Table 1, very few methods were designed and tested for large cell population tracking. Although very robust in their own applications addressing biological research, the methods proposed by [9] and [10] were designed for higher resolution cell tracking under fluorescence microscopy (essentially targeting cell nuclei, see S1). The approach of [12] is also designed for fluorescence microscopy and makes use of semi-supervised approach of dealing with errors (see S1). Besides being applied to fluorescence microscopy, the method developed in [27] is an online method (implemented as a microscope guiding macro) that moves the microscope stage to track individual cells. Although [1] handles unmarked cells, the spatio-temporal resolution used in the reported experiments does not allow to reach conclusions about the algorithm's robustness in tests with standard cell density environments. In a preliminary study [2] we presented a method that could track a large amount of cells and detect errors when a tracker drifted on a neighboring tracked cell. However, to be optimally efficient this error detection scheme requires that all cells be tracked. This was not the case in [2] because each time a tracker drift was detected one cell was lost, thereby continually decreasing the tracked cell count. In addition to these abandoned cells, new cells resulting from cell divisions or cells entering the observed volume after the tracker initialization step in the first frame were not tracked.

In light of all these observations, we propose an improved method that is designed to robustly follow large populations of unlabeled cells which are observed under phase-contrast microscopy through small magnification (i.e., 10×). This new method is described in detail in the following section and is then validated and applied on a real experimental application (see Results), before concluding with a discussion.

## Methods

### Principles of the new method

**Figure 1:**
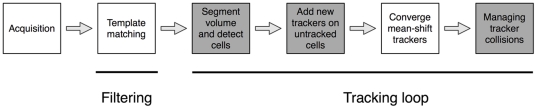
A schematic representation of the tracking algorithm steps. The gray boxes are significant improvements with regards to our preliminary work [2].

Although based on a preliminary study [2], the new method improves tracking performance by integrating automatic cell detection and tracker collision management (gray boxes in Figure 1), thereby reducing the risk of tracking errors. The cell detection step allows to track all cells and hence to recover those which were lost in [2], i.e., cells whose trackers drifted away or new cells that appear in the observed volume during the experiment. This translates practically into an increased number of tracked cells and an improved detection ability of tracker collisions, resulting in more accurate tracking results (see Results).

In our method we adopted the model evolution tracking paradigm, using classic mean-shift tracking [29] because of its simplicity (relatively few parameters to set up) and efficiency in tracking many small objects in a large volume. Each cell is assigned a single mean-shift kernel that is left to converge on the data from one time step to the next, thereby establishing the trajectories of its underlying cells.

Before starting the tracking, the entire sequence of volumes is preprocessed by a template matching step to smooth the volume artifacts in order to improve the convergence of the mean-shift kernels. Smoothing the volumes addresses the difficulties of low magnification and phase-contrast microscopy (i.e., gel and phase-contrast cell off-focus artifacts) and makes cell detection through segmentation possible.

Each preprocessed volume thus is segmented to detect untracked cells and assign them new mean-shift kernels, so to ensure all cells are tracked. Tracking all cells helps to solve the problem of kernel drifting onto a neighboring cell because it assures the detection of two kernels converging onto one cell. Furthermore, the segmentation allows to initiate tracking in the first image stack as well as of new cells that enter the volume later or arise from cell division, and thus constitutes significant improvement over the data reported previously [2].

Furthermore, an additional step of tracker collision management enhances algorithm robustness regarding cell oversegmentation and tracking difficulties encountered when two cells become too close.

Finally, we compute trajectory descriptors (e.g., cell speed and the largest traveled distance) which are normalized by the trajectory duration to allow comparison [16]. These descriptors are then used to compare the migration abilities of the studied cell populations subjected to different treatment conditions.

### Acquisition of volume sequences

**Figure 2:**
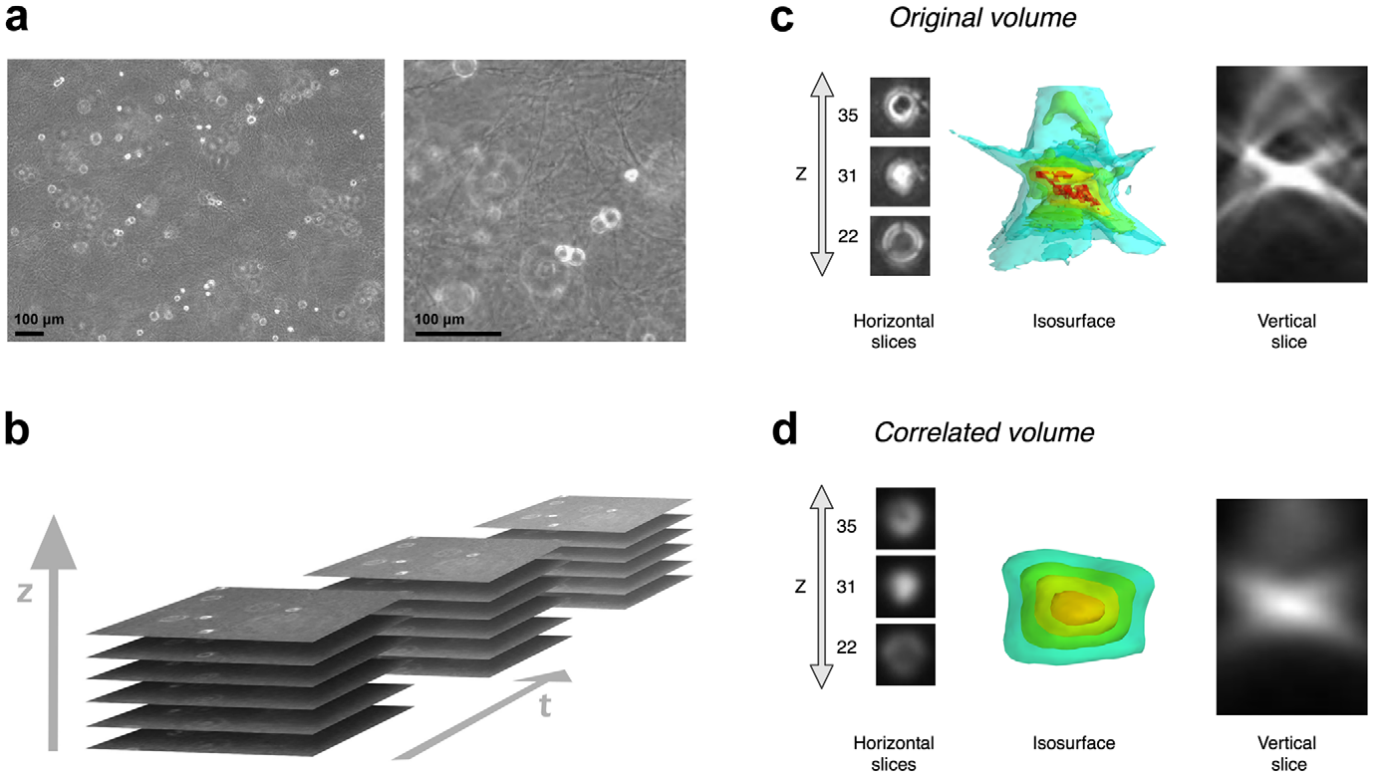
Cell appearance in 3D collagen gels. (a) Cell and collagen gel aspects in an original phase-contrast Z-slice with a zoom showing the collagen fibers. (b) Schematic presentation of the 3D time-lapse sequence acquisition. Detailed cell aspect in (c) the original phase-contrast volume (after contrast enhancement) and (d) the correlated volume, illustrated by several Z-slices, a XZ cut plane (vertical slice) and an intensity isosurface 3D view.

As previously detailed [2], the sequences of volumes in which we track the cells are produced in the following way. A 3D migration chamber [28], including cancer cells mixed in a collagen gel, is placed in a 37°C-heated incubator and is observed with a standard inverted phase-contrast microscope equipped with a 10× objective, a Z-motorized stage and a megapixel digital camera. Every 4 minutes during a period of 24 h to 48 h, a stack of 60 images is acquired, covering an observed volume with sides of 1250 µm×930 µm and 480 µm in depth, with an anisotropic resolution of 0.78 µm along the X and Y directions, and 8 µm along the Z direction (according to the depth resolution defined by the numerical aperture of the microscope objective). Figures 2a–b illustrate the acquisition process and the resulting image aspect.

### Algorithm outline and tracking method details

Initially, two preprocessing steps are performed to facilitate the rest of the tracking steps. First, we boost the contrast of the images to remove the background noise (mostly artifacts of the collagen gel that appear in phase-contrast microscopy, see Figure 2). Second and more importantly, each volume is correlated with a cell template (cf. *Correlation preprocessing* section). The correlation is essentially a template matching step that provides smoother intensity peaks coinciding with cells. The smoother gradient around the cell peaks render the mean-shift convergence more robust [2]. All the following steps (segmentation and tracking) happen in the correlated volume.

The tracking is initialized in the algorithm loop by detecting all cells. In fact, this detection stage is used at every time step to identify new (untracked) cells. The volume is segmented using a morphological soft maximum filter. All distinguishable intensity peaks, i.e., isolated cells or conjoined cells, are detected in this step (cf. *Segmentation* section). Each object detected as a cell is assigned a mean-shift kernel.

At every time-step *t*, the mean-shift kernels are left to converge in the current correlated volume from their previous position identified in the *t-1* volume, thereby establishing the cell trajectory (cf *Mean-shift tracking* section). Kernel drifting onto a neighboring cell can be detected when two kernels converge to the same point, which we call a “tracker collision”. Specific management rules allow to distinguish the case of cells touching from that resulting from cell oversegmentation, both causing tracker collisions (cf. *Detecting untracked objects and managing risks of tracking error* section). Detecting potential tracking errors due to cell proximity (very small distance between two or more cells) requires that all the cells be tracked in order that all tracker collisions, caused by the mean-shift drift of neighboring cells, are detected.

### Algorithm outline

Preprocessing step:

1. For a given microscopic setup, the user designates an isolated cell as the cell template
2. For every volume *I* perform correlation with the cell template to obtain the correlated volume *I_c_* (*Correlation preprocessing* section)

Tracking (in the correlated volume sequence):

1. If there are mean-shift trackers, let them converge in the current volume (*Mean-shift tracking* section)
2. Determine the trackers that converged to the same location (i.e., tracker collision) and remove both or only one (*Detecting untracked objects and managing risks of tracking error* section)
3. Detect all cells by segmenting the correlated volume (*Segmentation* section)
4. Add a mean-shift tracker on any cell that has been detected and does not have one yet (*Detecting untracked objects and managing risks of tracking error* section)
5. Load the next volume and jump to step 3 with all the trackers

### Correlation preprocessing

The cancer cells appear in 3D gels as small bright disks in their focal slice with growing off-focus phase-contrast interference rings. The 3D cell pattern consists in a pair of cones squashed into each other as illustrated in Figure 2c. The goal of the correlation is using the principle of template matching in order to eliminate the irregular phase-contrast artifacts around the cell and create a smooth gradient to aid the mean-shift convergence (*Mean-shift tracking* section).

Correlation was chosen over deconvolution (usual in fluorescence microscopy) because the latter is difficult in phase-contrast microscopy. The reason is that the phase-contrast images result from a non-linear optical process (merging both phase and intensity signals) for which no linear point spread function exists that could be used in a deconvolution process.

The correlation process is the following: a manually chosen subvolume cropped around an isolated cell is used as a template which is correlated with each volume of the sequence. In essence this correlation step transforms each original volume into a volume of correlation blobs, where the highest intensity level (i.e., correlation peak) inside each blob coincides with the center of the corresponding cell in the original volume (Figure 2d).

The following equation expresses correlation volume:
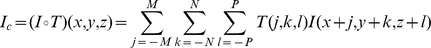
where 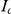 is the correlated volume, 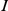 the original volume and 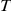 the template of size 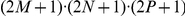. Such a correlation is computed using the Fast Fourier Transform algorithm.

Experiments previously reported in [2] showed that mean-shift convergence was not significantly influenced by the choice of the template used in the correlation step. This property also concern cells with dramatically different morphologies from the cell template because all cell patterns present relatively higher intensities on their central Z-slice. This point is further illustrated below in the case of an elongated cell. Other simpler templates were tested, such as 3D Gaussian balls, which did not filter out the phase-contrast rings as well as the cell template did. This is why that approach was not investigated further.

In practice this step is a preprocessing stage performed before the tracking and all correlated volumes are stored on disk.

### Segmentation

**Figure 3:**
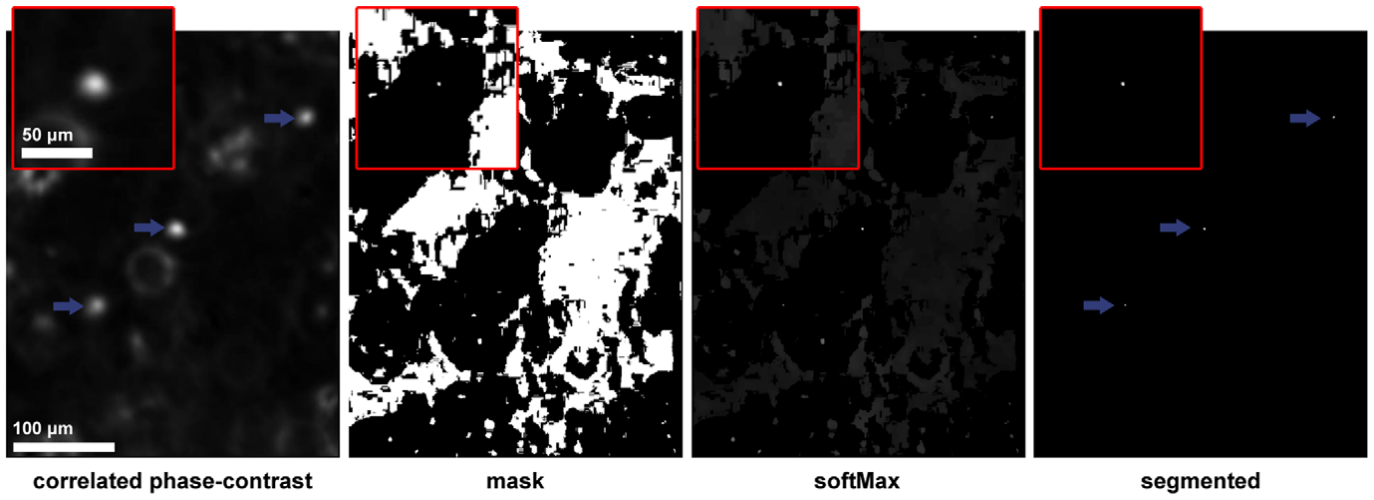
Cell detection in a correlated volume. Illustration of (a part of) a single Z-slice containing 3 cells in focus (pointed by arrows) submitted to the different segmentation steps (correlated phase-contrast, mask, softMax and the final segmented volume). The magnified region of interest shown in the upper left corner of each image is centered on the cell located in the middle of the image.

Detecting the cells is done in each correlated volume (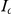) with a segmentation technique based on the local maximum filter. Detecting local maxima is necessary because the cell peak intensities vary in the correlated volume, making a simple thresholding inadequate. The different segmentation steps are illustrated in Figure 3.

First, the method performs a soft maximum filter on 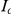 that results in a binary mask of all local maxima. This maxima mask is then applied to 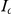. Finally, thresholding the masked 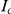 volume yields a binary volume where each cell is a distinct object.

A soft maximum filter is used to isolate the intensity peaks by setting to 1 the pixels that are close enough to their neighborhood's maximum intensity. It is applied to 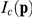 in the following three steps (where 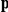 is the voxel coordinate vector in 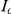):
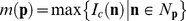
(1)

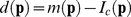
(2)

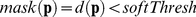
(3)The maximum filter (1) assigns the maximum intensity value of 3-D neighborhood, 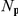, to its central voxel, resulting in 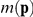. An anisotropic 3-D neighborhood (set to 20×20×10 voxels), which corresponds to a cell volume, is used to fit the anisotropic resolution of our data.

The *softThresh* in (3) is the softness of the maximum filter, i.e., the leniency (set to 5 in our 8-bit volume) with which an intensity value is considered to be close enough to its neighborhood's maximum. 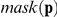 thus defines the regions where the intensities are close enough, i.e., within *softThresh*, to the maximum neighboring intensity.

By applying the *mask* to 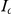 we obtain the *softMax* volume in (4), which contains all local maxima including low intensity ones present in the background. To distinguish these later from the actual cells in (5) we threshold *softMax* with *noiseThresh* (set to 120 in our 8-bit volume), dictated by the minimal cell intensity in 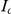.
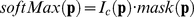
(4)

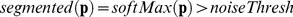
(5)The three parameters of the method are: neighborhood size 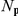, *softThresh* and *noiseThresh*. The latter two parameters have to be tuned with regards to the 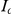 volume intensities as mentioned above. 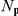 was set relative to cell size, so that any conjoined cells that are close enough to merge their trackers also appear as a single detected object. This approach allows us to detect correlation peaks corresponding to either isolated cells or conjoined cells. A detected peak defines a new object if there is no tracker on it (see *Detecting untracked objects and managing risks of tracking error* section). A cell tracker, i.e., a mean-shift kernel (see the following section), is then initialized on each new object.

### Mean-shift tracking

Mean-shift is an iterative procedure that seeks a local peak in a value distribution [29]. In a grayscale volume, such as a correlated volume, a mean-shift process converges to the nearest intensity peak from its initial position. By this way, the mean-shift process constructs the cell trajectory from one convergence point to the next one (in the next volume).

More formally, let *S* be a data set constituted by three-dimensional voxels, labeled **s**, weighted by their grayscale value, 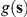. Let 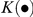 be a kernel, centered on the origin in the n-dimensional Euclidian space. The kernel mean is then defined as [29]:
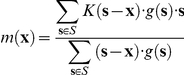
The mean-shift is defined as the difference 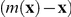, i.e., the shift of the kernel's current center towards its mean vector. The mean-shift is iterated until the kernel stabilizes, i.e., the kernel shift falls below a small distance (set to 0.01 voxel in our experiments). In our experiments, the tracking of a cell was automatically stopped when the cell center comes near the borders of the observed volume. The boundaries were fixed to 10 pixels in XY (2D slice) and 5 slices in Z.

For the present application, we use a flat kernel with a size (of 7 voxels) chosen relatively to the cell size and the time-lapse sampling interval. The average cell diameter observed on slice images is around 15 pixels (corresponding to about 20 µm) and the observed inter-frame cell speed is very rarely more than a cell radius. In addition, the 3D cell appearance covers 10 to 15 Z-slices. Our previous experiments in [2] showed that mean-shift convergence was not significantly influenced by small variations in the kernel size (3 to 9 voxels by side), whereas an over-sized kernel is prone to be attracted to adjacent cells.

### Detecting untracked objects and managing risks of tracking error

Tracking errors are automatically detected when two trackers merge or exhibit strong proximity. In this latter case, the two cell trajectories are stopped and the trackers removed because it is very difficult, even under user supervision, to detect with certainty which one is which. After that the volume is segmented to detect all the distinguishable objects (see *Segmentation* section). By labeling the segmented volume we can associate each tracker to its underlying label at that position. All the labels that do not have associated trackers are determined to be untracked. We then initiate a new trajectory on each untracked object by creating a new tracker in its geometrical center.

**Figure 4:**
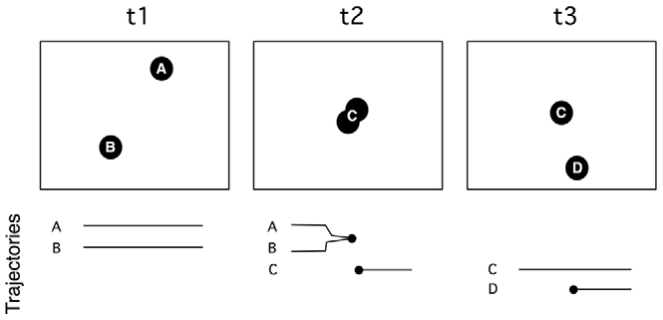
Schematic illustration of tracker management when two cell paths intersect. The timeline bellow the schematic frames presents the start and end points of the 4 trajectories obtained in this situation.

**Figure 5:**
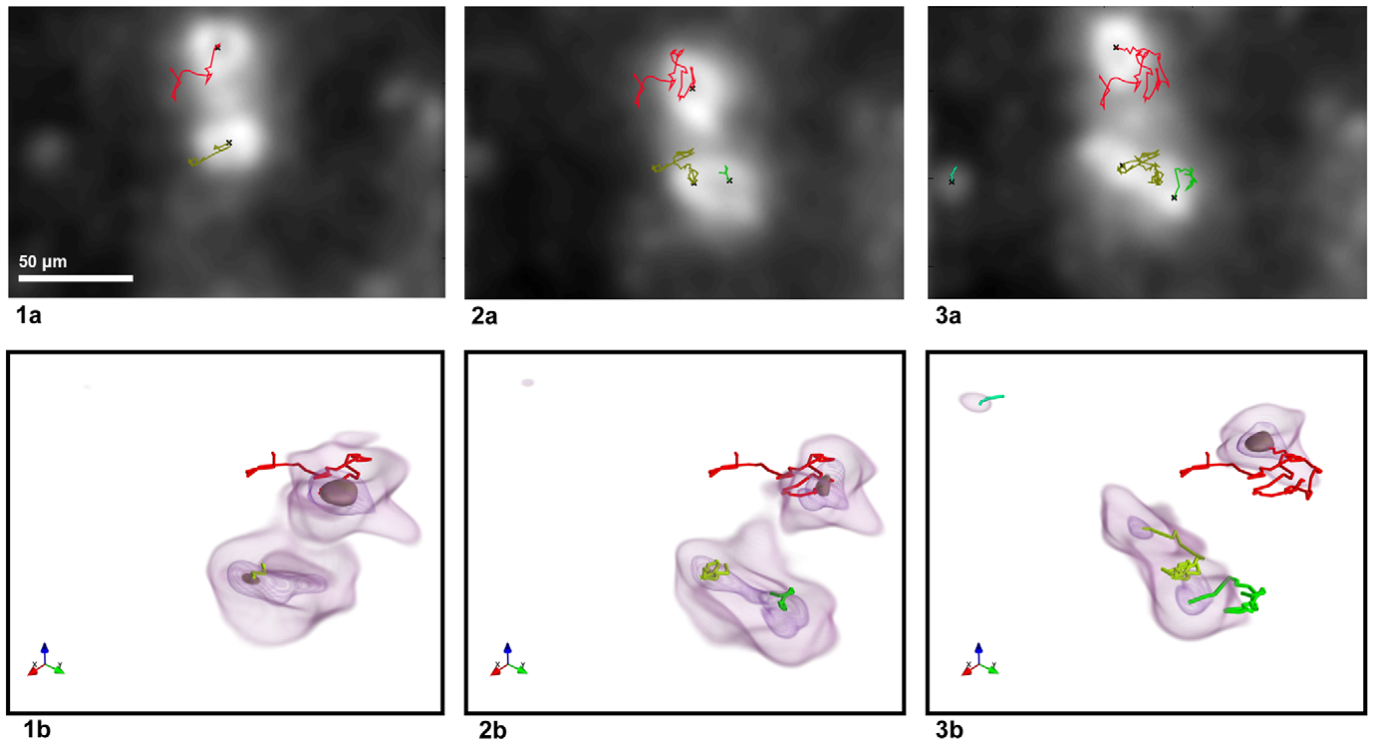
Detection of new cells in a volume sequence. The new cells result from (frame 2) a cell division or (frame 3) a cell entry into the observed volume. The trajectories are rendered on (1a–3a) the average intensity Z projections from the correlated volumes and (1b–3b) the corresponding intensity isosurfaces (3D view).

Figure 4 presents a schematized situation of two cells touching and parting. Initially at *t*1 two trackers (A and B) are correctly tracking their respective cells. At *t*2 the cells come too close and the trackers collide. As opposed to the initial method in [2] we do not automatically create a new tracker to start a sole trajectory there, but only stop the former two. The next step in the algorithm is the object detection. The newly conjoined cells are detected as one object and determined to be untracked, since there is no active tracker on it. A new tracker (C) is automatically created and will follow the conjoined cells as one. Some time later (*t*3) the cells part and the (C) tracker will stay on one of them. In the same loop, the other untracked cell will be detected and thus assigned a new tracker (D), thereby starting its own trajectory. The situation of dividing cells and cells entering the volume are handled similarly as illustrated in Figure 5 (frames 2 and 3 respectively).

**Figure 6:**
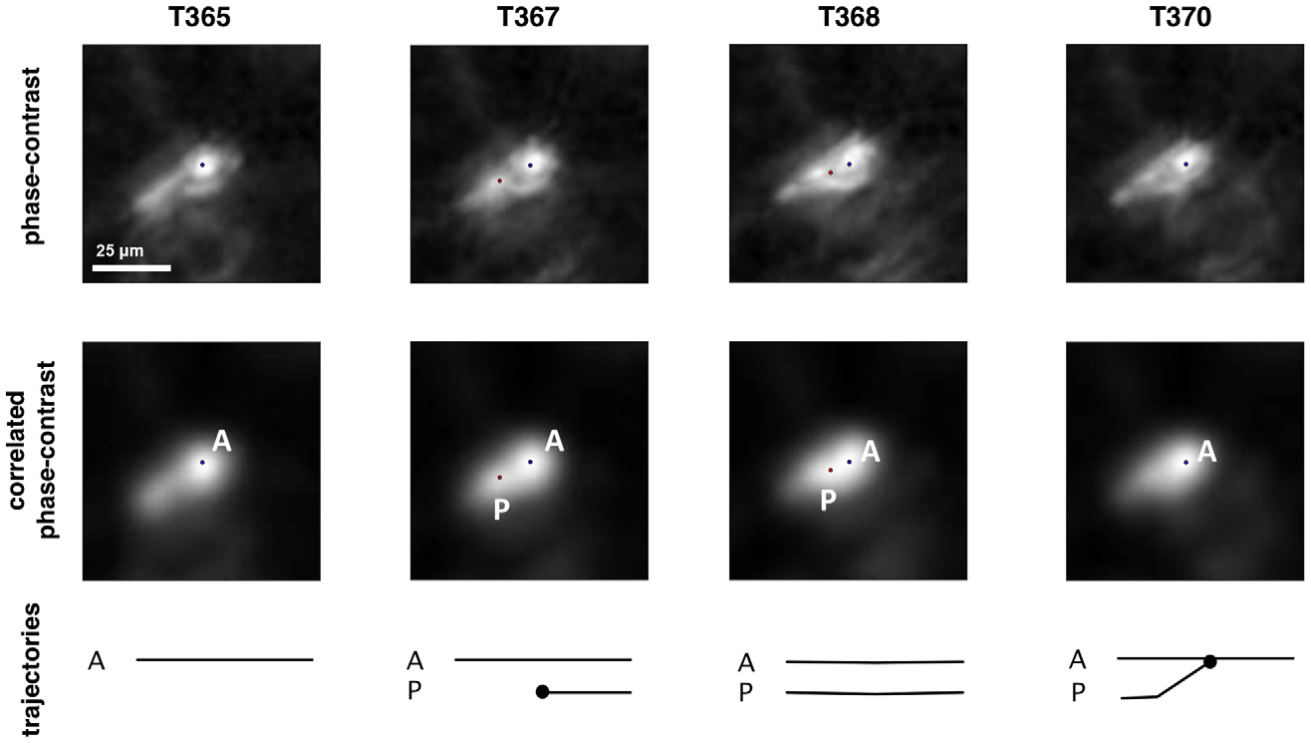
Tracking of an elongated cell. An elongated cell (blue dot) spawns a second parasite tracker (red dot) on a body extension (on the cell tail before its retraction when migrating). The secondary tracker is discarded as it converges onto the cell's original tracker within a couple of frames. The images show Z-slices centered on the cell in the phase-contrast (1st row) and correlated (2nd row) volumes. The timeline below the images is similar to that in figure 4.

However, we choose to unstop the two cell trajectories after the collision of their tracker if one of them presents an extremely short “lifetime” (1–5 time frames) when it collides with a tracker associated with a longer trajectory. The “short” tracker is considered to be a “parasite” and is removed without affecting the longer cell trajectory which keeps its tracker. These special situations can result from false cell detection or cell oversegmentation causing the erroneous initialization of a tracker near and immediately converging to an already tracked cell. As illustrated in Figure 6 a large number of these cell detection errors are automatically corrected by mean-shift convergence and the tracker collision management rules. This point is further illustrated in File S5 which shows the successful tracking of an elongated cell.

We also sometimes observe cells that move together, while remaining close to each other and causing a succession of trajectory intersections. These cases result in many short trajectory parts. These trajectories are often too short to be considered in the statistical analysis of the normalized features (see next section). Even though these short trajectories would not impact the statistical analyses, the cells are actually tracked and therefore allow us to detect potential collisions with others.

### Analyzing trajectories

When the tracking is done, we summarize each trajectory by features such as the maximum relative distance covered by the cell from its origin (MRDO) or the average cell speed [2], [30]. The value distribution of such a feature characterizes a cell population. The goal in migration studies is to confront the feature distributions of cell populations submitted to different experimental conditions and to detect significant differences among them.

The usual migration indicators, such as MRDO and average speed, are relative, i.e., they are distances normalized by the trajectory durations in order to make them comparable. The corollary is that short cell follow-ups increase the risk that the trajectory features are noisy and exhibit outlying values. To avoid this problem and to be biologically relevant for applications of drug screening, trajectories with too short follow-ups are filtered out by arbitrary fixing a minimal trajectory duration (i.e., at least 3h, i.e., 45 frames, of cell follow-up in our experiments). The chosen threshold allows to discard the outliers directly related to short cell follow-ups in the feature value distributions (data not shown).

## Results

This section begins by validating our new tracking approach on real biological sequences in 3D collagen gels. First, we validate the efficiency of the cell detection step. This first verification is important because the premise of our error detection step is that all cells are tracked. Second, we validate the complete tracking process by (i) showing that detecting (nearly) all cells improves automatic error detection with regards to our previous method [2] and (ii) quantifying the number of remaining tracking errors through manual supervision. Finally, we applied our approach on real biological experiments testing the impact of matrix components on cell migration abilities. All the analyzed sequences were recorded with the setup described above (see *Acquisition of volume sequences*).

### Cell detection validation and collision management

First, we manually identified 283 cells as test cells across 12 volumes and checked whether they were detected by our automatic phase-contrast based detection scheme. Of them, 27 (i.e., 9.5%) were not detected because their intensities in the correlated volume were below the detection threshold (c.f. noiseThresh in equation (5)). These weaker cell peaks are less prone to cause tracking errors because less able to attract a mean-shift kernel tracking a neighboring cell exhibiting a stronger peak. It should be noted that a cell being undetected at time *t* (because of its weak intensity peak) will be detected as soon as the signal intensity rises above the detection threshold.

Second, we validated that the detected objects by our method are indeed cells. We extracted the images of 366 objects detected as cells by our method and inspected them manually. All of them were confirmed to be cells.

**Figure 7:**
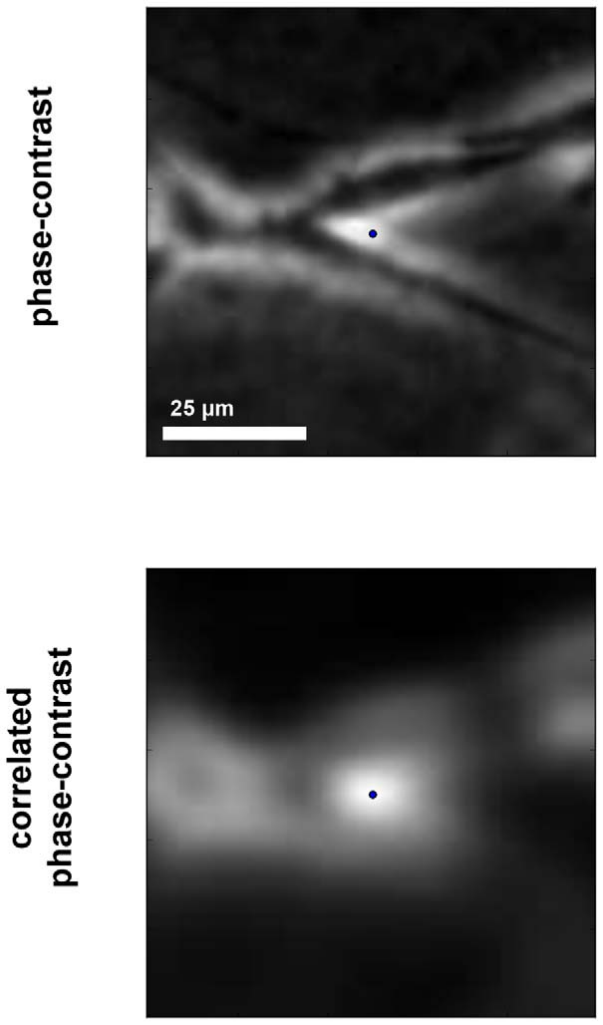
False cell detection due to phase-contrast interferences. The Z-slices of the phase-contrast (top) and correlated (bottom) volumes are centered on phase-contrast interferences falsely detected as a cell (blue dot). The actual cells are visible in the phase-contrast slice as dark elongations touching.

In a subsequent validation step, we monitored cases of cell tracker collision and observed very few detection errors. These errors either consist in cells detected twice or are due to artifacts occurring between neighboring cells. As illustrated in Figure 6, the situation where an elongated cell is detected twice is often and rapidly solved by the tracking process because the two peaks usually do not last long. This figure illustrates the best case where the secondary tracker (red dot) has a short trajectory before merging with the primary one (blue dot) on the cell body and is thus dealt as a parasite. In the worst scenario the secondary tracker has a longer life span and therefore prematurely stops the longer trajectory which correctly tracked the cell body. In rare other circumstances phase-contrast artifacts of two neighboring cells optically interact and reinforce the signal to create an intensity peak in the correlated volume at several Z-slices off focus. This peak is sometimes bright enough to be detected as a cell (see Figure 7). When the cells move away, the falsely detected tracker is attracted to one of the neighboring cells. This error is detected and the trajectories are stopped. Because these false trajectories are short they are either dealt as parasites or (if a bit longer) automatically filtered out the statistical analysis.

All this data shows that our new method is able to accurately detect cells in phase-contrast volumes as well as to correct detection errors by collision management.

### The new tracking method successfully tracks numerous cells and detects error risks

In this section we highlight the improvements brought by our new method with regards to [2]. More particularly, we show that the cell detection step strongly increases the number of tracked cells in a volume sequence. In addition to improve the statistical power of the extracted trajectory data, this increase also enhances the method ability to automatically detect close cells which risk to cause tracking errors. Finally, we globally validate our new method by quantifying its error rate based on the manual inspection of a subset of 100 trajectories.

#### Improvement of cell tracking quality due to the cell detection method

As quantified above, our cell detection method is very efficient and thus ensures that the tracker drifts are detected. In comparison, the absence of this detection step in [2] causes the systematic decrease of the number of tracked cells with each collision (due to the loss of one of the two interacting cells) in addition to missing new ones that appear in the observed volume after the first frame. All these aspects strongly reduced the capacity to detect collisions and therefore potential tracking errors.

To quantify the benefit of the new method, we tracked the cells in a given volume sequence and divided the obtained cell trajectories in two sets. Set1 is the set of the trajectories generated without using the additional cell detection step (like in [2]). It is thus constituted of the trajectories initialized in the first frame and those emerging from a collision between two Set1 trajectories (only one new trajectory was initialized after a collision). Set2 collects all the other cell trajectories which are initialized later in the sequence thanks to cell detection. There were 98 trajectories in Set1 and 507 in Set2.

**Figure 8:**
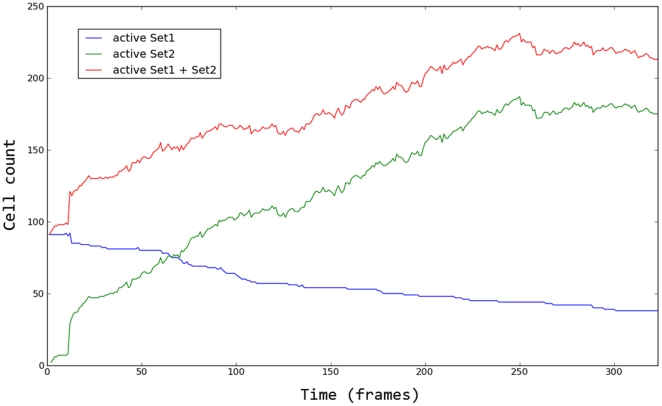
Number of active cell trajectories across a sequence. Set1 collects trajectories which are generated from the 1st volume without additional use of the cell detection step in the next volumes. Set2 collects all the other cell trajectories which are initialized later in the sequence thanks to this detection step. The graph shows the number of active trajectories (i.e., which are not stopped because of a collision with another cell tracker) in each set, and their union, over time.

Out of the 98 trajectories in Set1, 33 of them had a collision with a Set2 trajectory. These collisions represent 33 possible mean-shift tracker drifts that would be missed without the use of the segmentation step because only one of the two interacting cells was tracked. Throughout the sequence, these collisions progressively decreased the amount of unstopped trajectories in Set1 (i.e., those without risk of tracking error), as illustrated in Figure 8. This figure also shows that in Set2 the trajectories are initiated equally throughout the sequence, causing a progressive increase in the total number of tracked cells. These results clearly show the benefit brought by continuous cell detection in our new method.

#### Performance evaluated from a posteriori supervised trajectories

In a global way, we verify that the cell trajectories extracted by our new method have a low error rate by manually inspecting a set of automatically provided trajectories. We randomly took 100 trajectories from a pool of cell trajectories that were tracked for at least 3 h (long enough to be considered for statistical analysis). Based on the extracted cell trajectories, we generated a film centered on each cell position and inspected it manually to find whether the cell was correctly tracked. Examples of these movies are shown in supporting information and illustrate that our method successfully tracks cells, even in the case of a cell close to another (File S2). Supervision evidenced that only 6 trajectories of the 100 had errors. Those errors consist of either the tracker erroneously following an artifact (2 cases) or close cells incorrectly followed (4 cases).

### Real application

As an illustration of real applications of large cell population tracking, we present a biological experiment aiming to evaluate the impact of the matrix composition on brain cancer cell migration. Different studies have suggested that hyaluronic acid (HA) is a critical factor in the extracellular matrix of the brain for cancer cell invasion [31], [32] and could be a potential pharmacological target [33]. In order to evaluate the impact of HA on 3D migration of brain cancer cells, four experimental conditions of 3D collagen gels were set up with increasing proportion of HA, as described in Table 2, all supplemented with fetal calf serum (10%) and culture medium (48%). Human U373 glioblastoma cells (glioblastoma is one of the most invasive types of brain cancer) were mixed in these liquid gels before polymerization in 3D migration chamber, as previously detailed [2].

**Table 2:** 3D gel compositions in the different experiments.

Experiment name	Collagen (3 mg/ml)	Hyaluronic acid (3 mg/ml)
p42h0	42%	0%
p38h4	38%	4%
p30h12	30%	12%
p24h18	24%	18%

Collagen was provided by PureColTM (Nutacon, Netherlands) and hyaluronic acid was kindly provided by Auriga International (Braine-l'Alleud, Belgium).

**Figure 9:**
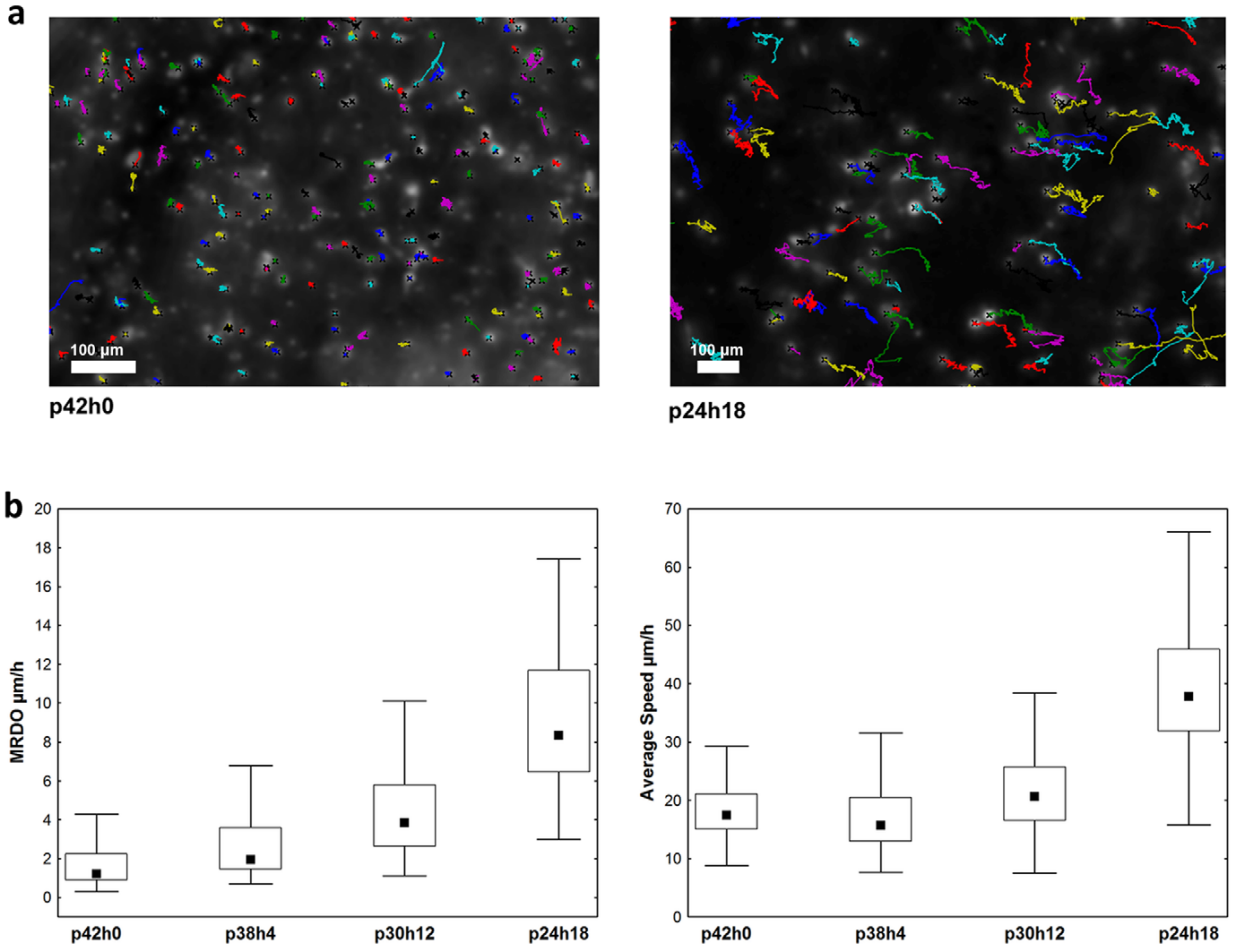
U373 cancer cell trajectories and the extracted features characterizing 3D migration behavior. (a) These trajectories are observed on the complete sequences in the absence (p42h0) or presence (p24h18) of 24% of HA in the collagen gel (b) The features (labeled on the Y axes) quantify the migration abilities of U373 cells in 3D gels which included a progressive increase of HA amount (see Table 2). The data distributions are presented by their median (square), inter-quartile range (box) and non-outlier range (bars).

The volume sequences (of 323 frames, i.e., about 1 day, for practical reasons) extracted from the different gel conditions were tracked using our new method. Figure 9a illustrates the trajectories obtained in control (p42h0) and an HA condition (p24h18). Further video illustrations of the latter condition can be found in File S3 and File S4. Trajectories longer than 3 h were used to extract the average speed and the MRDO features. Their distributions displayed per condition are shown in Figure 9b which evidence significant dose-response cell migration behavior (Kruskall-Wallis test: p<0.001). These data validate the expected pro-migratory effect induced on U373 cells by increased levels of HA in the cell microenvironments

## Discussion

We have presented a method that automatically and efficiently tracks large populations of unmarked cells in phase-contrast volume sequences. Our approach was motivated by the biologists' and pharmacologists' needs for a videomicroscopic assay in a 3D environment that would provide statistically relevant amounts of cell migration data. This is the reason why we opted for a method able to efficiently track individual cells in sequences of low magnification phase-contrast volumes.

Based on a previous work [2], the essential novelty of the presented method is the significantly improved robustness due to the addition of both continuous cell detection and tracker collision management that directly impact tracking quality. As described in the method, trajectories are successfully extracted until cells cross paths, whereby trajectory collisions are automatically detected and the trajectories are intentionally stopped to preserve validity (except in special cases of “parasites”). Manual tracking in low magnification sequences showed us that in a lot of tracker collision cases expert inspection could not discern trajectories (or did with great difficulty) after cells were mangled. There are probabilistic approaches that rebuild trajectory trees (e.g., [34]) from sets of partial trajectories. However we did not use such a trajectory post-processing method because we deemed that the improvement would not outweigh the added inaccuracy (based on reports of 80% accuracy in [9]).

Having been developed for labeled cells observed under confocal (or two-photon) microscopy, other methods using level set tracking [9], [10] appeared to be inappropriate in our use cases. The level-set methods define object boundaries, which are not clearly determined in phase-contrast volumes (due to the presence of off-focus artifacts). Furthermore, in situations of cell proximity, level-set approaches require accurate cell segmentation to avoid the level-sets from degenerating onto neighboring cells [10]. Such accurate segmentation is not possible in low resolution phase-contrast volumes and would thus cause error accumulation in a cell tracking process.

Another alternative approach, combining cell segmentation and inter-frame association [1], relies on flawless object detection and thus requires very sparse cell seeding to be efficient. Failing to detect a cell or cell oversegmentation at a given time-step would directly impact the inter-frame association, causing tracking errors. Including cell features into the inter-frame association can help improve association robustness as far as these cell features exhibit a certain level of inter-frame stability [12]. No such stable feature was identified in our correlated volume sequences. We project to extend this kind of investigation in initial phase-contrast volumes (see below), as well as on the extracted cell trajectories, in order to possibly identify stable cell features. These features could enable tracker collision management to be improved in case of cell crossing, i.e., without requiring trajectory stopping.

In our approach, the detection step in correlated volumes is only used to initiate mean-shift trackers when none are present. Our detection scheme does not provide flawless detection: cell intensities may fall bellow detection threshold, two cells in close proximity may be detected as one object or one cell may be detected as two objects. We solved these problems by using a mean-shift process, which robustly follows the cell intensity peaks and thereby performs inter-frame association, combined with tracker collision management in order to extract valid cell trajectories.

In view of the quality of the obtained trajectories, it is possible to extract small 2D image sequences centered on a single cell migrating in a 3D environment (as illustrated in Files S5, S6 and S7). This method output allows biologists to easily observe cell behavior as well as cellular morphology and their variations with time. One of our future research goals is to automatically extract and analyze the cell shape and its dynamics to enrich the quantitative comparison of cell population behavior with morphological descriptors. Even though cell segmentation is a complex task in 3D phase-contrast image stacks, we can reasonably expect that the 3D cell center locations provided by our tracking method should strongly help to extract cell boundaries and thus morphology features. In this way, we could also identify stable cell features which would allow to improve cell tracking, as mentioned above in this discussion.

Our tracking method could also be adapted to analyze volumes acquired with digital holography microscopy (DHM). This relatively new microscopy technology is able to produce volumes similar to the phase-contrast microscopy ones, without requiring optical Z-sectioning, thanks to a computer-based in depth refocusing ability [3]. A sample hologram recorded with a CCD camera is numerically reconstructed to provide a stack of slice images refocused at incremental depths. The main advantages are a reduction in the amount of data to be stored, the time required to record the full 3D information and strong Z-resolution improvement [3]. Studies have shown that DHM enables marker-free 3D cell tracking [3], [4]. However, this technology is not widely used yet and requires tests and validation in the context of robust automatic tracking of large cell populations moving in 3D volumes.

Finally, our algorithm was developed in Python using its standard scientific libraries. In the present version, the total processing time (on a modern desktop computer) of one volume with hundreds of trackers is around 2 minutes, which is half of the 4 minutes time-lapse step used for acquisition. A refactored version of our source code is under development and is not yet publicly available. We will publish it under a free software license as soon as it is ready for public use (with an API and usage examples).

## Supporting Information

File S1 **The detailed analysis of the related works summarized in** **Table 1** **.**
(DOC)

File S2 **Video of tracked cells showing trajectories when cells cross paths and then part (Z projection computed using the mean intensity).**
(AVI)

File S3 **Video of a tracked volume sequence (Z projection computed using the mean intensity).**
(AVI)

File S4 **Video of trajectories rendered in a volume (the aspect ratios of the volume size are not respected).**
(AVI)

File S5 **Video showing volume (dimension in voxels: 100×100×5) centered on tracked cells, allowing the observation of the cell morphology (Z projection computed using the mean intensity).**
(AVI)

File S6 **Video showing volume (dimension in voxels: 100×100×5) centered on tracked cells, allowing the observation of the cell morphology (Z projection computed using the mean intensity).**
(AVI)

File S7 **Video showing volume (dimension in voxels: 100×100×5) centered on tracked cells, allowing the observation of the cell morphology (Z projection computed using the mean intensity).**
(AVI)
