# Supplementary material for: A New Method to Address Unmet Needs for Extracting Individual Cell Migration Features from a Large Number of Cells Embedded in 3D Volumes
Source: PLoS One. 2011 Jul 15;6(7):e22263. doi: 10.1371/journal.pone.0022263 (PMC3137636; doi:10.1371/journal.pone.0022263)
Supplement: File S1 — The detailed analysis of the related works summarized in Table 1 . (DOC) [file pone.0022263.s001.doc]

### Supporting information S1

**Comparison of works proposing 3D cell tracking approaches**

The analysis of related works detailed below supports the data summarized in Table 1 (in the main text). It confronts each method with different criteria in relation with the needs dictated by the drug screening application in focus in the present paper.

**Regarding cell labeling and microscopy technology**

To our knowledge, the only studies that concern tracking of unlabeled cells in 3D gels are [1] and [2], whereas the other methods track labeled cells under fluorescence-based microscopy including sophisticated and costly confocal or two-photon technology [9,10,12]. While these later are techniques of choice to carry out biological research, they are less adapted for drug screening applications because of their cost. Indeed, these applications require that multiple acquisition systems run in parallel on different treatment conditions (involving different molecules at different concentrations) to ensure result validity in comparative experiments. Additionally, cell labeling requires to verify that the resulting cell manipulations did not interfere with the study goal [SUP1, SUP2]. In particular, genetic modifications, such as the insertion of a fluorescent fusion protein, may affect the mechanism under study in biological research or the drug mechanism of action in drug screening, a mechanism which can be unknown when new molecules are analyzed. As mentioned in the introduction, fluorescence-based microscopy is also faced with cell toxicity, marker fading and problematic marker transfer during cell division.

While the above data suggest that unlabeled cells observed under contrast enhancement microscopy seems better adapted in the context of drug screening applications, this approach makes more challenging the subsequent image analysis task required for the analysis of cells migrating in 3D gels. Indeed, the image stacks acquired from a 3D gel do not present the same characteristics under the two types of microscopy and thus require different techniques of image processing. A well known example is image deconvolution which is usual in fluorescence microscopy [6] and is difficult in phase-contrast microscopy because this latter results in a non-linear optical process for which no linear point spread function exists.

The use of fluorescent probes provides different advantages. More specifically, fluorescent probes targeting cell nuclei (as used in [9, 10]) ease (i) cell tracking and cell division handling because of the less complex shape of nuclei, comparatively to whole cells, and also (ii) distinction between close cells since nuclei are more spatially isolated than external cell boundaries. However, nucleus labeling requires genetic modifications (insertion of a fluorescent fusion protein targeting a nucleus protein) which increase the risk of a biological impact. In comparison, fluorescent dyes or nanoparticles (respectively labeling cell membrane or cytoplasm) need simple cell incubation only. Under fluorescence microscopy acquisition, the common difficulties in image analysis consist in managing the possible variations in the fluorescent labeling aspects during the cell life as well as a low signal-to-noise ratio, i.e. the difficulty is to correctly detect cells/nuclei form the background noise [9,10].

In the case of contrast enhancement microscopy, the absence of cell/nucleus labeling ease both cell culture preparation and acquisition steps. However, the difficulties increase in image analysis because (i) cell (and nucleus) boundaries are less delimited, (ii) polymerized gels usually generate refringent patterns in the volume background and (iii) contrast enhancement creates artifacts, such as phase interference rings generated by phase-contrast around each cell in its neighboring off-focus z-planes (as illustrated in Figure 2 in the main text).

**Regarding magnification and observed volume size**

All the methods described in Table 1 (cf. main text) are created to track cells and to analyze their trajectories. However, the focus on the data can be different. Most of the studies mentioned here [9,10,12,27] were developed and tested with images acquired under high magnification (63x). This enables a small number of cells to be observed at a higher spatial (x-y) resolution. Consequently, the reduced size of the observed volume makes it difficult to follow the same set of cells for a long time, because cells may rapidly exit the field of view whereas others may enter in it. For example, the real datasets analyzed in [10] are constituted of stacks with a maximum size of 147 x 147 x 5 µm, knowing that a cell diameter is about 20 µm. The advantage of the approach in [27] is that as the microscope stage is moved for each cell individually, they are not limited by the initial bounds of the observed volume, like all the other offline methods.

A higher spatio-temporal resolution is more appropriate for extracting cell shape and also finer analyses of cell behavior such as cell-cell interaction, as it is specifically mentioned as the study goal in [12]. These specific aspects usually require suitable cell labeling and fluorescence microscopy to better identify cells.

In contrast, the later two studies [1] and [2] use a grosser spatio-temporal resolution, in particular [1] (mag 10x, at least 15 z-slices every 20µm, 1 frame/30min) whereas [2] enhanced the Z and time resolution (mag 10x, 60 Z-slices every 8µm, 1 frame/4min). Both studies focus only on cell migration without considering cell morphology. The sparse sampling carried out in [23] increases the risk of tracking errors. This is why these later authors used 3D cell cultures made with very low cell seeding density. Complying with this constraint decreases the probability of close cells being miss-associated across consecutive frames using their nearest neighbor association tracking, thereby giving rise to erroneous trajectories. In comparison, the higher resolution used in [2] strongly helps to better following of cells and provides statistical robustness when large cell populations are tracked.

**Considerations of the tracking technique**

There are two main paradigms for tracking cells in 3D sequences. The first is frame-by-frame image segmentation tracking. It consists in first detecting object candidates in each frame and then performing an inter-frame correspondence of each object, thereby establishing its trajectory. In the case of [23] the segmentation is done by thresholding and the association by a nearest-neighbor algorithm. A refined approach is used in [12] by involving mean-shift segmentation to yield accurate segmentation of the cells. In addition, the temporal association step uses a multiple hypothesis method, whereby an object is associated with several probable instances of itself in the next frame. At the end of the sequence the best (more probable) path combination is chosen for each cell.

The second paradigm is a model evolution approach which consists in optimizing a parameterized model for each object to be tracked and using it as tracker. Usually an initialization step based on segmentation sets the model on each cell at the beginning of the sequence and lets it evolve with time to the subsequent cell positions. This approach has as advantage that each object is identified by its tracker. Both [9] and [10] used a level set function for modeling each cell, so defining the cell shape or, more often, the nucleus shape, depending of the marker used for cell labeling (as mentioned above) [9,10]. The level set function obtained in one time step is let to evolve on the image of the next time step, thereby adopting the new object position and shape.

Another type of model involves a local mode-seeking process, such as a mean-shift kernel which moves along a gradient in a signal to finally converge in the nearest local intensity peak. In a preliminary study [2], we used a combination of template correlation and mean-shift. While the template correlation creates an intensity peak at the center of each cell, the mean-shift process converges from a cell’s location at one time step to its location in the next.

The method presented in [27] is a microscope software script and thus can only be used online: it moves the stage in XYZ to center on each tracked cell in order to recompute the cell’s center iteratively at each time step by using a custom method. Even thought the acquisition time is optimized, the number of tracked cells directly influences the possible temporal resolution.

**Management of special events and detection of potential tracking errors for improving tracking robustness**

An important aspect of automatic tracking methods is a robust handling of special cell events and the detection of potential errors. A majority of tracking errors arise when cells come into close proximity causing mix-up between them.

The algorithms that were developed for higher resolutions detect object morphology. Such methods, as in [9,10], manage naturally events like cell proximity and cell splitting, through their formulation of level sets, which allow objects splitting but not merging. Furthermore, in [10] specific measurements are applied to cells detected as being in close proximity in order to further automatic preventions of errors.

A different approach adopted in [12], which tracks two types of cells using different cell labels, consists in detecting potential errors in segmentation and tracking and presenting them to the user for correction. The error detection is based on confronting extracted object characteristics (such as cell volume) to experimental data distributions previously validated. The cases identified as being outliers in those distributions are considered to be potential errors. The user is presented with these cases to manually correct confirmed errors.

Finally, strong cell proximity that leads different cell trackers to erroneously converge onto a single cell is automatically detected in [2]. The tracking of the affected cells is then stopped to safeguard the validity of the trajectory data. A new tracker is created at the convergence point to follow one of the cells, whereas the other(s) is (are) lost.

The remaining problem of new cells entering the observed volume during the experiment is more easily managed by frame-by-frame image segmentation combined with an adapted inter-frame correspondence method (such as in [1, 12]). Model-based technique requires to prepare cell models in stand by near volume boundaries [9]. However, this approach requires additional constraints to avoid that new models intersect with any of the existing cells, leading to incorrect segmentation results [10]. Entering of new cells is not tackled in [2].

**References not mentioned in the main text:**

SUP1. Boddington SE, Sutton EJ, Henning TD, Nedopil AJ, Sennino B et al. (2010). Labeling Human Mesenchymal Stem Cells with Fluorescent Contrast Agents: the Biological Impact. Mol Imaging Biol.. In press. DOI: 10.1007/s11307-010-0322-0

SUP2. Jaiswal JK, Simon SM. (2007) Optical monitoring of single cells using quantum dots.

Methods Mol Biol 374: 93-104.
